# Can a combined screening/treatment programme prevent premature failure of renal transplants due to chronic rejection in patients with HLA antibodies: study protocol for the multicentre randomised controlled OuTSMART trial

**DOI:** 10.1186/1745-6215-15-30

**Published:** 2014-01-21

**Authors:** Anthony Dorling, Irene Rebollo-Mesa, Rachel Hilton, Janet L Peacock, Robert Vaughan, Leanne Gardner, Guilherme Danzi, Richard Baker, Brendan Clark, Raj C Thuraisingham, Matthew Buckland, Michael Picton, Susan Martin, Richard Borrows, David Briggs, Robert Horne, Paul McCrone, Joanna Kelly, Caroline Murphy

**Affiliations:** 1 MRC Centre for Transplantation, King’s College London, Guy’s Hospital, Great Maze Pond, London SE1 9RT, UK; 2 Department of Nephrology and Transplantation, Guy’s Hospital, Great Maze Pond, London SE1 9RT, UK; 3 King’s College London, Capital House, 42 Weston Street, London SE1 3QD, UK; 4 Renal Unit, St James’s University Hospital, Beckett Street, Leeds LS9 7TF, UK; 5 Transplant Immunology, Level 09 Gledhow Wing, St James’s University Hospital, Beckett Street, Leeds LS9 7TF, UK; 6 Renal Unit, The Royal London Hospital, London E1 1BB, UK; 7 Clinical Transplantation Laboratory, The Royal London Hospital, 2nd Floor, Pathology & Pharmacy Building, 80 Newark Street, London E1 1BB, UK; 8 Department of Renal Medicine, Manchester Royal Infirmary, Oxford Road, Manchester M13 9WL, UK; 9 Transplantation Laboratory, Manchester Royal Infirmary, Oxford Road, Manchester M13 9WL, UK; 10 Renal Unit, University Hospital Birmingham, Edgbaston, Birmingham B15 2LN, UK; 11 NHSBT Birmingham, Vincent Drive, Edgbaston, Birmingham B15 2SG, UK; 12 Centre for Behavioural Medicine, UCL School of Pharmacy, University College London, London WC1H 9JP, UK; 13 King’s Clinical Trials Unit, King’s College London, Institute of Psychiatry, PO64, M2.06, London, UK

**Keywords:** Human leukocyte antigen antibodies, renal transplantation, premature allograft failure, chronic rejection, screening, tacrolimus, mycophenolate mofetil, clinical trial, randomised controlled trial, randomized controlled trial, KCTU, CTU, trials unit, diabetes, kidney, graft failure, biomarker, biomarker-led care, DSA, non-DSA, multicentre, immunosuppression, usual care, treatment as usual, standard care, blinded, unblinded, EME funded, NIHR, optimised, optimized, intention-to-treat, per protocol

## Abstract

**Background:**

Renal transplantation is the best treatment for kidney failure, in terms of length and quality of life and cost-effectiveness. However, most transplants fail after 10 to 12 years, consigning patients back onto dialysis. Damage by the immune system accounts for approximately 50% of failing transplants and it is possible to identify patients at risk by screening for the presence of antibodies against human leukocyte antigens. However, it is not clear how best to treat patients with antibodies. This trial will test a combined screening and treatment protocol in renal transplant recipients.

**Methods/Design:**

Recipients >1 year post-transplantation, aged 18 to 70 with an estimated glomerular filtration rate >30 mL/min will be randomly allocated to blinded or unblinded screening arms, before being screened for the presence of antibodies. In the unblinded arm, test results will be revealed. Those with antibodies will have biomarker-led care, consisting of a change in their anti-rejection drugs to prednisone, tacrolimus and mycophenolate mofetil. In the blinded arm, screening results will be double blinded and all recruits will remain on current therapy (standard care). In both arms, those without antibodies will be retested every 8 months for 3 years. The primary outcome is the 3-year kidney failure rate for the antibody-positive recruits, as measured by initiation of long-term dialysis or re-transplantation, predicted to be approximately 20% in the standard care group but <10% in biomarker-led care. The secondary outcomes include the rate of transplant dysfunction, incidence of infection, cancer and diabetes mellitus, an analysis of adherence with medication and a health economic analysis of the combined screening and treatment protocol. Blood samples will be collected and stored every 4 months and will form the basis of separately funded studies to identify new biomarkers associated with the outcomes.

**Discussion:**

We have evidence that the biomarker-led care regime will be effective at preventing graft dysfunction and expect this to feed through to graft survival. This trial will confirm the benefit of routine screening and lead to a greater understanding of how to keep kidney transplants working longer.

**Trial registration:**

Current Controlled Trials ISRCTN46157828.

## Background

The problem addressed by this study is premature transplant failure – kidney transplants do not last for the natural lifespan of most recipients. Premature in this context refers to the lifespan of the recipient^a^. Current death-censored 10-year transplant survival rates vary between 59% and 70%, so 30% to 40% of patients have their transplant for <10 years [1]. Since 2000, a consistent annual attrition rate of around 3% of kidney transplants [2] means that approximately 700 patients return to dialysis each year in the United Kingdom. Although many of these patients are eligible for a second transplant, the legacy of the first often makes it harder to find a well-matched second kidney. In addition, second (and any subsequent) transplants have a shorter lifespan than the original transplant, so the problem of premature failure becomes amplified. Of the various reasons why transplanted kidneys fail, the single most common cause is immune-mediated injury [3].

Two types of study have linked antibodies (Ab) against human leukocyte antigen (HLAs) to immune-mediated injury and premature graft failure. Case–control studies have compared patients who have lost grafts with those in whom grafts are still working, performing retrospective analysis of prospectively collected serum samples. For instance, Mizutani *et al*. [4] studied 39 patients with failed grafts due to chronic rejection (CR) and 26 matched controls with functioning grafts. In the former group, 72% had immunoglobulin G (IgG) HLA Ab, compared to 46% of controls. Similar results were obtained from a different study of a separate population [5]. The surprising thing from these studies was the high incidence of HLA Ab in patients with working grafts. There are several potential explanations for this. It is possible that factors relating to the HLA Ab (such as complement fixing ability) or factors in addition to the Ab, influence the progression of CR and thus the timing of eventual graft rejection. A second, related possibility, is that all patients with HLA Ab develop pathology, but it progresses at different rates, such that patients showing up in the control groups in these studies are deteriorating more slowly. Evidence for this comes from Mizutani *et al*. [4], who showed that their CR group with HLA Ab showed progressive deterioration of renal function prior to graft failure. The same progressive deterioration was seen in the control group of patients with HLA Ab, whose grafts did not fail. These data illustrate that CR is a time-dependent process in which progressive graft dysfunction precedes graft failure. Moreover, the time from development of HLA Ab to graft failure is highly variable in different people.

Separate studies have reported the prospective follow-up of outcomes in those with HLA Ab. Terasaki and Ozawa [6] studied 2,231 patients. In a group of 479 with HLA Ab, the 2-year graft failure rate was 15%, compared to 6.8% in the 1,753 with no HLA Ab. This trial noted that the patients who failed within 2 years had worse renal function on testing than those that did not, consistent with the fact that CR is a progressive and time-dependent process and those that fail are at the end of this process. In another study, the same group [7] reported that the 4-year survival rate for 1,329 patients, all with functioning transplants, was 58% for those with HLA Ab (158 patients) vs 81% for those without (806).

Lachmann *et al*. [8] performed the best study to date. The study was based at a single centre in Berlin and had 1,014 patients with stable kidney function (for the 6 months pre-recruitment) and were on average 6 years post-transplantation. Patients were tested for HLA Ab and prospectively followed for 5½ years. Grafts failed in 37% of the 302 who had HLA Ab, but in only 17% of the 712 patients who tested negative for HLA Ab. Moreover, in this latter group, a subgroup of 195 patients had a repeat test performed 2½ years into the study. Of these, 148 remained negative and only 6% of grafts failed in this group. In contrast, 47 had developed new HLA Ab since the beginning of the study and 21% of these suffered graft failure, confirming that the development of new HLA Ab in the negative group was predictive of future graft loss. Finally, this study identified a difference between the prognostic value of HLA Ab that were specific for the donor, known as donor-specific antibodies (DSA), which were found in 33% of HLA Ab + patients, and those that were not (non-DSA), found in 66%. Graft failure rates were 51% over 5.5 years in patients with DSA and 30% in patients with non-DSA. In a subgroup of patients who had transplant biopsies, 78% of those with failed grafts and HLA Ab + had changes consistent with CR. They concluded that grafts in patients with HLA Ab were more than three times likely to fail than those without, even when corrected for age, sex, year of transplant, estimated glomerular filtration rate (GFR) and number of previous kidney transplants. These findings have been corroborated by a second study from the Netherlands [9], in which the risk of graft failure with HLA Ab was also shown to be independent of graft dysfunction and proteinuria.

Therefore, the literature indicates that HLA Ab are a prognostic biomarker and could be used as a screening test to identify patients at high risk of premature kidney transplant failure. All transplant units in the United Kingdom have the ability to detect HLA Ab, but routine systematic post-transplant screening of patients has not been generally adopted because it is not clear how best to treat patients once identified. Although there is a widespread view in the literature that HLA Ab cause CR, there is no evidence of this in humans. The chief investigator’s group has been investigating patients with CR for several years and has evidence that the activity of T and B cells is most strongly associated with progression of CR and that this subgroup benefits from enhanced immunosuppression (Shiu *et al.*, manuscript in preparation). This study will therefore test a combined screening and treatment protocol for HLA Ab in a randomised controlled trial.

## Methods/Design

### Trial objectives

#### Primary objective

The primary objective is the 3-year graft failure rate for patients testing positive for HLA Ab at baseline or within 3 years of randomisation who receive an optimised anti-rejection medication intervention with prednisone, tacrolimus (tac) and mycophenolate mofetil (MMF) (treatment), compared to a control group who test positive for HLA Ab at baseline or within 3 years post-randomisation who remain on their established immunotherapy and whose clinicians are not aware of their Ab status.

#### Secondary objectives

Determine the 3-year graft failure rate in patients randomised to unblinded HLA Ab screening, compared to a control group randomised to blinded HLA Ab screening.

Determine whether the treatment influences patient survival.

Determine whether the treatment influences the development of graft dysfunction as assessed by the presence of proteinuria (protein/creatinine ratio (PCR) > 50) or a change in the estimated glomerular filtration rate (eGFR).

Determine whether the treatment influences the rate of acute rejection for these groups.

Determine the adverse effect profiles of treatment in this group, in particular whether they are associated with increased risk of infection, malignancy or diabetes mellitus (DM).

Determine the cost-effectiveness of routine screening for HLA Ab and prolonging transplant survival using this screening and treatment protocol.

Determine the impact of biomarker screening and the treatment on the patients’ adherence to drug therapy and their perceptions of the risk to the health of the transplant.

Collect regular samples from patients enrolled in the HLA Ab + groups to enable an in-depth scientific analysis of the HLA Ab and cells of the immune and repair systems and to develop more sophisticated biomarkers for progression and/or responsiveness to therapy (to be funded separately, not as part of the efficacy and mechanism evaluation (EME) programme).

### Primary end points

The primary end point is the 3-year graft failure rate in HLA Ab + patients. Graft failure will be defined as restarting long-term dialysis or requiring a new transplant. Time zero starts at the time of randomisation, once the result of the HLA test is known. For those patients who are HLA Ab– at randomisation but become Ab + during a subsequent screening, time zero will be reset at the time of the positive test, and the follow-up time will be extended to allow for the 3-year end point.

### Secondary end points

The secondary clinical end points are:

• 3-year graft failure rates in all patients recruited to the trial

• Patient survival

• Rate of progression of graft dysfunction, as assessed by the change in the degree of proteinuria (PCR > 50) or the change in estimated GFR over 3 years

• Rate of biopsy-proven rejection over 3 years

• Rates of culture- or polymerase chain reaction-positive infection, biopsy-proven malignancy and DM

• Health economic analysis of outcomes

• Analysis of adherence and perceptions of risk for the biomarker-led care (BLC) groups

• The secondary scientific end points are:

• Changes in characteristics of HLA Ab and laboratory parameters of T and B cell phenotypes and responsiveness for the HLA Ab + groups

• Change in numbers and phenotype of circulating CD34+ cells for the HLA Ab + groups

## Trial design

**Figure 1:**
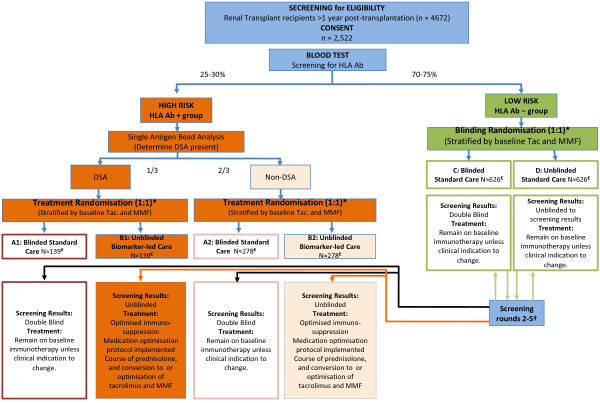
**OuTSMART flow diagram.** *Randomisation is performed using the results of a recruit’s first screening test. Those with donor specific antibodies (DSA) undergo no further screening as part of the trial (but serum will be stored for analysis of human leukocyte antigen antibody profiles later). †Those initially HLA Ab– undergo routine screening every 8 months. There is no second randomisation. A recruit allocated to blinded standard care (group C) who becomes HLA Ab + (black lines) will remain in a standard care group (group A1 or A2). If in the unblinded standard care group (D), they will change to unblinded biomarker-led treatment care (group B1 or B2) (orange lines). ‡ The numbers in each group are those anticipated at the primary end point. Ab, antibodies; DSA, donor-specific antibodies; HLA, human leukocyte antigen; MMF, mycophenolate mofetil; tac, tacrolimus.

This is a prospective open-labelled randomised marker-based strategy trial, with a standard control, where biomarker stratification is based on HLA Ab status. The biomarker-strategy design is appropriate for testing the clinical utility of a biomarker [10]. Recruitment will take place in five British renal transplant units over 3 years, with recruits followed for at least 3 years. The trial design is shown in the flow diagram in Figure 1, which shows the number of patients anticipated to be in each group by the end of the trial, based on sample size calculations, consent rates, eligibility and estimated attrition. Recipients of cross-match negative transplants aged 18 to 70, >1 year post-transplant with eGFR ≥ 30 will be asked to give consent for the screening/treatment process.

The first stratification will be based on blood test screening for HLA Ab. Approximately 30% will be HLA positive, with approximately 70% negative. HLA Ab + patients will be further screened with single-antigen beads (SAB) to determine whether DSA are present (approximately will have 1/3 DSA and 2/3 non-DSA). Thus, biomarker stratification has three arms (DSA+, non-DSA + and HLA Ab–). The second stratification will be based on current immunosuppression to ensure balanced numbers already on tac or MMF in each group. The immunosuppression stratification has four groups: MMF only, tac only, both tac and MMF, and neither tac nor MMF. The third stratification factor will be the recruiting study site (five sites).

**Figure 2:**
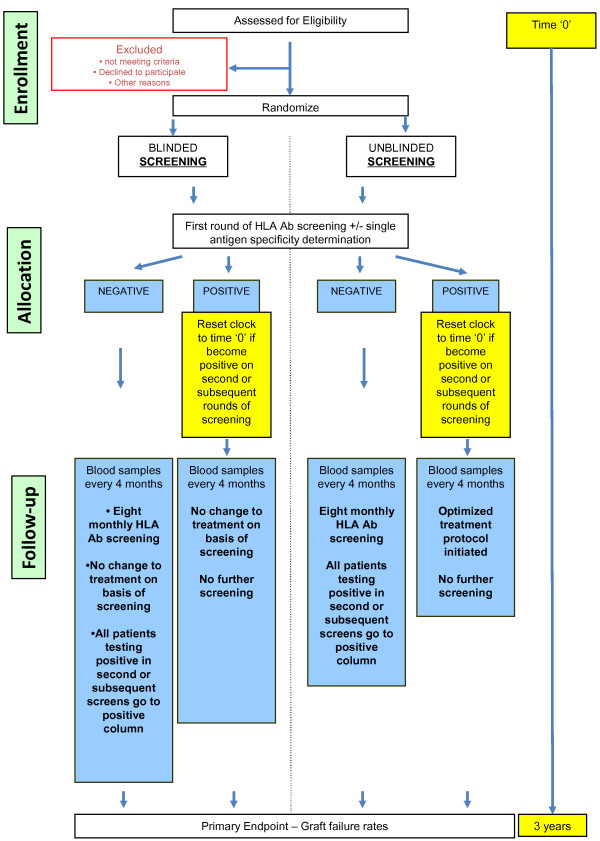
**Consort diagram for OuTSMART.** Ab, antibody; HLA, human leukocyte antigen.

HLA Ab + patients will be randomised 1:1 into either blinded standard care (SC) or unblinded BLC. Patients in the former (groups A1 and A2 in Figure 1) will be blind to their biomarker status and will remain on baseline immunotherapy, whereas the clinicians of patients in the latter (groups B1 and B2 in Figure 1) will know their HLA Ab status and will be offered treatment. HLA Ab– patients will remain on their existing immunotherapy and randomised 1:1 into either the blinded (C) or unblinded group (D), with only the latter knowing their HLA Ab status. Both these groups will receive regular Ab status monitoring for the first 3 years. Those patients who become positive during subsequent screening rounds (approximately 10% per year) will be moved to the appropriate HLA Ab + groups (DSA + or non-DSA+) for final data analysis. All patients in group D found to be positive on the second or subsequent rounds will be offered the same treatment as those patients who were positive in the first screening round, and followed up for 3 additional years from the time they become positive. Thus, the maximum amount of time any single patient can remain in the study is 6 years. ‘New’ patients, randomised but initially HLA negative at baseline, will be included in the intended primary analysis of HLA positive groups at each successive 8 monthly screening round. The patient flow diagram according to CONSORT guidelines is provided as Figure 2.

### Sample size calculations

The primary purpose of this trial is to demonstrate superior outcomes using a defined treatment strategy compared to standard care for biomarker (HLA Ab) positive patients. Once superiority is demonstrated for HLA Ab + patients, it is also necessary to demonstrate non-inferior outcomes when comparing the unblinded screening strategy to the blinded standard of care for the entire patient population, including those HLA Ab–. The graft failure rate 3 years after screening has been chosen as a clinically relevant primary outcome. As a reference for power calculations, we have used the observed failure rates reported by Lachmann *et al*. [8] for HLA Ab + and HLA Ab– patients. Since failure rates differ between DSA + and non-DSA + patients, sample size calculations have been carried out separately for these groups. Following these calculations, we estimated the number to be screened, based on expected dropout rates, expected screening results and eligibility criteria (see below). We based our estimates of the differences in primary outcome between the groups on, first, the results of our preliminary data from patients with CR treated with a similar regime as used here, and second, our assessment that large differences in primary outcome will be needed to make the screening programme cost-effective.

Hypothesis testing will be sequential [11]. Superiority will be tested first in the HLA Ab + groups to demonstrate the efficacy of the treatment. If this analysis is significant, then non-inferiority will be tested for the entire population to demonstrate the clinical utility of the biomarker screening. We calculated the sample size needed to show the superiority of the treatment optimisation for biomarker-positive patients using the hypotheses as follows.

Hypothesis 1.1: HLA Ab + patients with DSA, randomised to standard care (A1) will show higher graft failure rates than patients randomised to biomarker-led care (B1).

We hypothesise that the experimental treatment will bring the failure rate of group B1 down to that of non-DSA patients in standard care (A2). Assuming a 30% failure rate for group A1 (as in [8]), and a 16% failure rate for group B1, 139 patients per group will provide 80% power and 5% type I errors, for a two-sided test.

Hypothesis 1.2: HLA Ab + patients, with non-DSA, randomised to standard care (A2) will show higher graft failure rate than patients randomised to biomarker-led care (B2).

We hypothesise that the experimental treatment will bring the failure rate of group B2 down to that of biomarker negative patients in standard care (C). Assuming a 16% failure rate for group A2 (as in [8]), and a 6% failure rate for group B2, 146 patients per group will provide 80% power and 5% type I errors, for a two-sided test.

The numbers enrolled in groups A and B include those patients initially enrolled in groups C or D who become HLA Ab + during re-screening.

We calculated the sample size needed to show the non-inferiority of all unblinded patients compared to all blinded patients as follows.

Hypothesis 2: All patients randomised to unblinded screening (combined groups B1 + B2 + D) will show equal or lower graft failure rates than all patients randomised to blinded screening (combined groups A1 + A2 + C), irrespective of biomarker status.

At the end of the trial, we expect 60% of patients to be in the HLA Ab– groups, 13% in DSA + groups and 27% in non-DSA + groups (after dropouts). Assuming a 6% failure rate in biomarker negative groups C and D, the estimated average failure rate of all blinded screening patients is 11.8% (that is, (6 × 0.6) + (16 × 0.27) + (30 × 0.13)). Similarly, the estimated average failure rate in the unblinded screening group is 7.3%. We have established a non-inferiority limit of 5% absolute difference, so that the unblinded group would be considered non-inferior with a failure rate of 16.8% or lower. For this purpose, 1,043 patients in each arm would provide 90% power and 2.5% (one-sided) type I errors.

We will keep recruiting until every group has acquired the number required to test both hypotheses. When calculating the number of patients to screen for eligibility and to recruit, the following assumptions were made: (1) 60% of patients under follow-up at each transplant centre will meet the eligibility criteria, (2) 10% will refuse consent, (3) the prevalence of HLA Ab in most transplant populations is 25% to 30%, (4) 6% of initially Ab– patients will become Ab + in each screening round and (5) 2% will drop out every screening round.

Based on these assumptions and a recruitment audit in participating centres, we have estimated that we need to assess eligibility for 4,672 patients and enrol 2,522 to ensure sufficient numbers are in each group at the primary end point to test both hypotheses. Figure 1 shows the number of patients needed in each of the arms to reach the primary end point to test hypothesis 2, which is sufficient to test hypotheses 1.1 and 1.2 (the numbers in groups A and B include those patients initially enrolled in groups C or D who were found to be HLA Ab + during re-screening).

A recruitment audit has revealed that there is a very high chance of recruiting all the required patients from five participating centres within 3 years. We anticipate recruiting sufficient numbers of patients to all groups by screening round 3 or 4. Each centre is expected to recruit similar numbers of patients.

### Statistical analysis

#### Primary analysis

Statistical analysis will be on an intention-to-treat and treatment-received basis. We will consider the patients who become positive during the follow-up in the appropriate group.

The proportion of graft failures for each of the groups is denoted by π_A1_, π_B1_, etc.

Superiority (hypothesis 1):

H_0_: π_A1_ = π_B1_ and π_A2_ = π_B2_

H_1_: π_A1_ ≠ π_B1_ and π_A2_ ≠ π_B2_

To test superiority for the primary outcome for the biomarker (HLA Ab) positive groups (hypotheses 1.1 and 1.2), we will use a two-sided *Z*-test at the 5% level of significance, and will calculate the 95% CI for the difference in the proportion of graft failures between the biomarker-led care and standard care groups. The biomarker-led care groups will be considered superior if the absolute *Z* > 1.96, and our assumptions with respect to the size of the differences will supported if the CI includes a positive difference in proportions of 14% for hypothesis 1.1, and 10% for hypothesis 1.2.

Non-inferiority (hypothesis 2):

H_0_: π_Unblind_ – π_Blind_ ≥ δ

H_1_: π_Unblind_ – π_Blind_ < δ

To test for non-inferiority of the unblinded groups compared to the blinded groups (hypothesis 2), we will carry out a one-sided *Z*-test at the 2.5% level of significance. We will conclude non-inferiority if H_0_ is rejected, and the corresponding upper bound of the 95% CI excludes the limit δ (set at 5% more failures in the unblinded groups).

#### Secondary analysis

A similar procedure will be followed for secondary binary outcomes. We will use Cox proportional hazards regression to analyse survival outcomes. For continuous secondary outcomes we will use a two-sample *t*-test, transforming data where they are skewed.

### Exploratory moderator analysis

Additionally, for all primary and secondary analyses, we will carry out multivariable analyses, using generalised linear models, to adjust for the effects of stratification factors (for example, the previous immunosuppression regime). In this case, we will use the corresponding odds ratio to evaluate the adjusted size of the differences between proportions, by transforming back the estimated odds ratio associated with the treatment group into absolute differences in proportions. If the results of the analysis of the primary outcome are significantly affected, the adjusted effects will be taken as final.

### Handling missing data

Where a given outcome is measured at two or more time points, missing post-randomisation assessments will be dealt with by fitting linear mixed models to all the available measures using maximum likelihood methods. Such an approach provides valid inferences under the assumption that the missing data mechanism is ignorable (or Missing at Random). If post-randomisation variables are found to be predictive of the dropout rate, multiple imputation will be considered using *K*-nearest neighbours.

### Economic evaluation

Cost data is usually skew but will be analysed using arithmetic means so that total costs are preserved. Non-normality in errors will be allowed for by using generalised linear models with an appropriate error structure (for example, a gamma distribution [12]). Incremental cost-effectiveness ratios or an incremental cost-utility ratio will be presented where appropriate. Cost-effectiveness acceptability curves will be plotted to summarise the uncertainty in cost-effectiveness.

### Detailed study plan

#### Selection of subjects

The local transplant clinic database will be used to identify patients meeting the baseline inclusion and exclusion criteria. At the start of the trial, the entire population of transplant clinic attendees who meet the eligibility criteria are potentially eligible for recruitment. On subsequent screening rounds, patients who reach 12 months post-transplantation after the start of the trial will become eligible and these will be recruited before the next screening round.

#### Informed consent

Potentially eligible patients will be approached at a routine clinic appointment by the principal investigator (PI) or research nurses and given printed and verbal information about the trial. They will have the opportunity to return for a second consultation within a few days to give informed consent for recruitment into the study or to do this on their next routine appointment. Alternatively, eligible patients will be sent information about the study through the post, for discussion and consent at their next routine appointment. Following consent, full eligibility criteria will be reviewed. This may include testing for chronic viral diseases (if they have not been tested within the last 5 years) and pregnancy (if a patient’s history suggests there is the possibility of pregnancy).

#### Inclusion criteria

• Sufficient grasp of English language to give written and witnessed informed consent to participate

• Renal transplant recipients >1 year post-transplantation, male or female

• Aged 18 to 70 years

• Estimated glomerular filtration rate (eGFR) ≥ 30, based on the four-variable Modification of Diet in Renal Disease (MDRD) approach

#### Exclusion criteria

• Recipient requiring HLA desensitisation to remove antibodies for a positive cross-match transplant

• Recipient known already to have HLA antibodies who has received specific intervention for those antibodies or for CR

• Recipient of an additional solid organ transplant (for example, pancreas, heart, etc.)

• History of malignancy in previous 5 years (excluding non-melanomatous tumours limited to the skin)

• Positive for hepatitis B surface antigens, hepatitis B core antibodies, hepatitis C or HIV (according to a test performed within the previous 5 years)

• History of acute rejection requiring escalation of immunosuppression in the 6 months prior to screening

• Patient enrolled in any other study involving administration of another investigational medical product (IMP) at time of recruitment

The following exclusion criteria are based on the summaries of the product characteristics of the IMPs:

• History of an ongoing or previous infection (no time limit) that would prevent optimisation of immunosuppression, including ocular herpes simplex

• Known hypersensitivity to any of the IMPs

• Known hereditary disorders of carbohydrate metabolism

• Pregnancy or breastfeeding females (based on verbal history of recipient)

• Pre-menopausal females who refuse to consent to using suitable methods of contraception throughout the trial

### Randomisation procedure and code break

Prior to randomisation but after consent, site staff will register a recruit on the web-based electronic data capture system (InferMed MACRO), hosted at the King’s Clinical Trials Unit (KCTU). Each will be assigned a unique study patient identification number (PIN) by the system. Samples from all recruits will be sent to the HLA laboratory, along with this PIN and a sample request form containing other information required for randomisation.

Laboratory staff will screen for HLA Ab and perform SAB testing on positive screening samples to check for the presence of DSA. Once this information is known, the lab staff will access the KCTU randomisation system and randomise the patient, using the HLA Ab results. Randomisation will be further stratified by centre and current immunosuppression. The lab staff, PIs and nurses at the site will be automatically emailed and the randomisation system will inform them whether the patient is in a blinded or unblinded group. If the patient is in an unblinded group, the system will inform the PI of the HLA Ab status. Unblinded patients will be identified by blue stickers appended to the notes and all future clinical samples. The system will tell the trial staff to enter HLA Ab– patients into the subsequent 8-monthly screening rounds, and also whether the patients have been selected to provide future samples for the 4-monthly scientific analysis (for transfer to the chief investigator’s laboratory). This information will be relayed using a star on the blue labels that are appended to the laboratory request forms.

The notes and samples from blinded patients will have green stickers and labels. The HLA Ab status will not be given to the PIs or trial staff. A star will be used to tell the trial staff which recruits have been selected to provide 4-monthly samples for transfer back to the chief investigator’s lab for scientific analysis. These patients will have samples taken every 8 months for HLA Ab screening. Once inside the lab, the lab staff will use their knowledge of the HLA status to determine which HLA Ab– patients will undergo screening. The samples from HLA Ab + patients will be discarded.

On the second and subsequent HLA Ab screening rounds, the lab staff will update the randomisation system. The results from patients in the unblinded groups only will be forwarded to the PI and laboratory staff via email. This will indicate whether the status has changed, which will trigger the change in the treatment protocol for those that have changed from HLA Ab negative to positive.

Laboratory staff at each recruiting site, with access to HLA Ab results, will be provided with a unique username and password to access the randomisation system. Password access must be authorised by the trial manager in all cases and direct requests from sites will not be processed. Access to the system is via the internet [13] by clicking the ‘randomisation – advanced’ link and selecting the OuTSMART Trial.

There are no blinded study medications in the trial so no emergency code break is required. In the event that a study site clinician wishes to be made aware of blinded laboratory results, this must be discussed and agreed with the trial manager and the chief investigator in all cases. It is not anticipated that unblinding in this manner will be required and only in extraordinary circumstances would this be agreed.

### Trial medication

All treatments will be introduced on the basis that they will be tailored to the individual patient, according to compliance, tolerance and achievement of target levels (for tac). Failure to tolerate one or more of the components of the protocol (or refusal to take any of the agents) will not be used as a reason for withdrawal from the study.

#### Investigational medical products and dosing regimen

The optimised treatment protocol in the two groups (B1 and B2 in Figure 1) with HLA Ab will be:

• Mycophenolate mofetil twice, three times or four times per day, or enteric-coated mycophenolic acid twice per day, with the daily dose determined according to local unit guidelines. The patient will be stabilised on the maximum tolerated dose.

• Tacrolimus once or twice per day, according to local unit preference, with dose titrated to achieve 12-hour post-dose levels of 4 μg/L to 8 μg/L (4 ng/mL to 8 ng/mL). The patient will be stabilised on the maximum tolerated dose that achieves these levels.

• Prednisolone once per day. Starting at 20 mg for two weeks, then reducing by 5 mg once per day every two weeks down to their previous maintenance dose or 5 mg once per day.

After consultation with the Medicines and Healthcare products Regulatory Agency (MHRA), we have confirmed that all these medicines will be classed as IMPs, whereas all others will not. Mycophenolate mofetil/mycophenolic acid is being used outside of its marketing authorisation (which states that it should be used with ciclosporin). However, because it is now used widely in combination with tacrolimus in most units in the UK, the two can be regarded as standard care. We therefore propose that the three drugs will not require labelling in line with annex 13 of the Rules Governing Medicinal Products in the European Union. This means the IMPs can be managed as normal, through a general practitioner or hospital prescription (as appropriate) and will not require special labelling, accountability, storage, etc.

#### Concomitant medication

Patients in all groups will have blood pressure controlled and total cholesterol lowered, using agents according to local unit guidelines and working to unit-defined targets. All other medication and treatment will be determined by local unit guidelines.

### Withdrawal of subjects

Individual recruits can withdraw at any time for any reason. Failure to tolerate one or more components of the treatment will definitely not be seen as a reason to withdraw an individual participant from the trial but is to be anticipated as an integral part of individualising therapy. Recruitment of new participants to the study will be halted temporarily on the advice of the data monitoring and ethics committee if any of the following are noted: a patient death attributable to treatment or an unacceptable incidence of severe adverse events attributable to treatment (if occurring in >10% of patients). In both these instances, the trial will undergo urgent review by the data monitoring and ethics committee and the trial steering committee.

The investigator has the right to withdraw patients from treatment with the study drug in the event of inter-current illnesses, adverse events, severe adverse events, suspected unexpected serious adverse reactions, protocol violations, cure, administrative reasons or other reasons. It is understood by all concerned that an excessive rate of withdrawals can render the study uninterpretable and unnecessary withdrawal of patients should be avoided. Should a patient decide to withdraw from the study, all efforts will be made to report the reason for withdrawal as thoroughly as possible. Should a patient withdraw from treatment with the study drug only, efforts will be made to obtain follow-up data, with the permission of the patient.

Participants who wish to withdraw from treatment will be asked to confirm whether they are still willing to provide study-specific data and samples for scientific laboratory analysis according to the trial protocol.

### Expected duration of trial

The trial is expected to recruit for 3 years. We estimated that the minimum number of individuals in each of groups C and D still HLA Ab– at the primary end point should be 627 (see Figure 1). The recruitment target is to randomise 950 patients to each of these groups. This allows for the predicted dropout rate and the rate of antibody conversion with a margin of error. Patients will be followed up for a period of 3 years post-recruitment, except for patients in groups C and D who become Ab + during the initial 3 years of follow-up, who will transfer to the relevant Ab + group and be followed up for a further 3 years from the date of transfer. Therefore, the maximum amount of time that any single patient can remain in the study is 6 years. Communication of the need for an extended follow-up will be made at the final HLA screening round at month 32, after the primary outcome data have been collected, to avoid unblinding the HLA status in the blinded care arm.

### Trial procedures

#### Synopsis

A structured screening programme for IgG HLA Ab is proposed in patients who provide consent. Results obtained will initially be blinded to the transplant clinicians and patients. Patients will be randomised through the King’s Clinical Trials Unit and stratified: (1) DSA+, non-DSA + or HLA Ab–, (2) by current immunosuppression (tac alone, MMF alone, both tac and MMF, or neither tac nor MMF) and (3) recruiting study site. Patients in groups A1, A2 and C (see flow diagram in Figure 1) will remain blinded to the results of their screening (as will their clinicians), whereas those in B1, B2 and D will learn whether they are HLA Ab + or Ab–. All recruits will undergo a final test for HLA Ab status as they reach the end of the study. The optimised treatment protocol for patients in the two groups with HLA Ab (B1 and B2) is outlined above. Patients in all groups will have blood pressure controlled and total cholesterol lowered, according to local unit guidelines.

#### First visit

Post consent, patients who have not been screened for HIV or hepatitis B/C within the last 5 years will undergo additional screening tests for these viruses. Female patients who report they may be pregnant will have a blood test for beta-Human Chorionic Gonadotrophin levels. Once eligibility criteria have been met, the following baseline data will be recorded at recruitment:

• Weight

• Blood pressure

• Sex

• Ethnicity

• Age

• Date of birth

• HLA type

• HLA type of donor kidney (if known)

• Any significant past medical history, including history of diabetes mellitus, cause of renal failure, details of previous transplants and cause of graft loss, evidence of sensitisation pre-transplantation (panel reactive antibody and antibody specificities if known)

• Medication including doses

• PCR of urine sample

All patients will then have blood taken for;

• Baseline clinical parameters: full blood count (minimum haemoglobin, white cell count, platelets); biochemical series (creatinine, Na^+^, K^+^, bicarbonate, calcium, C-reactive protein, lipid profile, glucose); MDRD eGFR on latest creatinine; current calcineurin inhibitor 12-hour trough levels (as appropriate); total immunoglobulin levels

• Scientific analyses: 50 to 60 mL blood for separation of peripheral blood mononuclear cells (PBMC) and 20 mL for serum storage

• Analysis of HLA Ab status (10 mL clotted blood), as described above, which will allow randomisation to proceed

All patients will be asked to complete questionnaires to assess attitudes to risk and adherence.

#### Subsequent visits

The optimised treatment protocol will be introduced within the first 3 months in those HLA Ab + patients allocated to this group. Recruits will be seen up to every two weeks during this period (a maximum of six extra clinic appointments are envisaged), though they should be on a maintenance dose of prednisolone 7 weeks after initiating optimisation. During this period they will have full blood count (as above), creatinine, Na^+^, K^+^, glucose, calcineurin inhibitor trough levels and blood pressure monitored according to the trial protocol. Once stabilised, they will be seen at least every 4 months in the transplant clinic. Patients allocated to all other groups will be seen at least every 4 months in the transplant clinic for formal study assessments. Patients may be seen at other times during this period, according to clinical need, but study assessments should be done within the time parameters established in the protocol.

Once every 4 months the following will be recorded:

• Weight

• Blood pressure

• Full blood count (minimum haemoglobin, white cell count, platelets)

• Biochemical series (creatinine, Na^+^, K^+^, bicarbonate, calcium, C-reactive protein, glucose)

• MDRD eGFR on latest creatinine

• Calcineurin inhibitor trough levels

• PCR of urine sample

• Total immunoglobulin levels

• Episodes of infection, malignancy or new DM

• Episodes considered to be adverse events

Every 12 months, each patient’s lipid profile will be measured.

For all patients with HLA Ab, and a small cohort of patients without, separate blood samples will be taken for non-routine scientific analyses as above. Steps will be taken to ensure this sampling does not break the blinding of group allocations. Once every 8 months, HLA Ab– patients will undergo further screening for HLA Ab (see above). At the end of the study, all patients will undergo a final test for HLA Ab status.

In recruits with living donors (known to them), the living donors will be invited to participate either by the recruit or directly by the study team, following consent from the recruit to inform donors of their participation in the study. Donors will attend the clinic at their convenience, where consent will be taken and blood (60 mL) taken for separation of PBMC, which will be stored in the research laboratory, identified only as from the donor of a particular study recruit. Donors may be asked to donate another aliquot of blood at another time within the next 3 years.

### Laboratory tests

#### Human leukocyte antigen antibody analysis

Serum prepared from 10 mL of blood will be used in the commercially available LABScreen tests (One Lambda, Canoga Park, CA), containing fluorescently tagged beads coated with purified HLA antigens. All participating centres have Luminex equipment (Luminex Corp, Austin, Texas) for analysis of these tests and the skills to process samples and interpret results. Therefore, the tests will be performed in each of the centres. A sequential analysis of samples is planned, first to identify those with HLA Ab, using mixed class I and class II Ab screening beads coated with multiple different types of HLA. The manufacturer’s definition of positive and negative tests will be used when interpreting the results. In those patients with positive results, the specificity of the HLA Ab will be determined by SAB, coated with single HLA class I or class II antigens. As before, when interpreting the results, the manufacturer’s definition of positive and negative tests will be used. Any patient with a positive test for HLA Ab identified by SAB will be regarded as HLA Ab + for the trial if the mean fluorescence intensity (MFI) is ≥2,000. If the HLA Ab are directed against a mismatched donor HLA antigen, the patient will be assigned as DSA+. The number of DSA with MFI ≥ 2,000 will be used to define the Ab ‘burden’ of an individual. In the final analysis, correlations between HLA Ab burden and outcomes will be sought. Patients with SAB positivity that is difficult to label as DSA + or non-DSA + (because of insufficient data on donor mismatches, for instance), will be regarded as being non-DSA+. Patients with a positive reaction on screening but lacking reactivity with the SAB at the level described will be considered negative. Excess serum will be stored. The same screening will be undertaken for HLA Ab– patients every 8 months, with the samples taken at a routine clinic appointment.

#### Routine biochemical, haematological and calcineurin inhibitor trough analysis

These will be performed by the local clinical laboratories and the results recorded as above.

#### Scientific laboratory analysis

A laboratory analysis will be performed at recruitment for all patients and then every 4 months on patients with HLA Ab and a small cohort of patients who are HLA Ab–, chosen randomly at randomisation. Then, 60 to 80 mL blood will be taken and 20 mL for serum. The samples will be sent to the research laboratory of the chief investigator where the blood will be separated into PBMC and CD34+ cells. DNA and RNA will be extracted from a small number of cells and stored. All other cells will be stored in aliquots in liquid nitrogen until thawed for analysis. Serum samples will be stored at -80°C until thawed for analysis. The same process for preparing PBMC will be used for the samples from donors.

Patients will also consent to analysis, for research purposes, of any stored serum, blood or tissue (such as transplant biopsies). This will apply for all existing and future samples taken for clinical reasons. In the case of future transplant biopsies, the patients will be asked to consent to having an extra core taken for transcriptome analysis. Subject to available funding, this will be stored in an appropriate medium and transported to the chief investigator’s laboratory for storage.

### Procedures for assessing efficacy parameters

Graft failure is defined as the return to long-term dialysis or re-transplantation. This will be measured from the date of recruitment and will be reported for failure due to any cause. The date of restarting dialysis or re-transplantation will be recorded on the Case Report Form.

Deteriorating renal function will be determined by linear regression analysis of the serial MDRD eGFRs from the point of recruitment (like that used by Dudley *et al*. [14]). Deterioration is defined as a negative slope. To be valid, the linear regression should yield an adjusted *r*^2^ ≥ 0.35 and have *P* ≤ 0.05 for the slope value. Stable renal function will be defined as serial MDRD eGFRs that are not significantly different from the horizontal baseline (that is, *P* > 0.05 on linear regression analysis). An improvement in renal function will be defined as a change in the slope so that it becomes positive. Serum creatinine levels and MDRD eGFR for the 12 months preceding recruitment and for the whole time patients are on the trial will be recorded on the CRF.

Proteinuria is defined by the PCR of a urine sample and is significant if >50. The PCR will be recorded on the CRF.

Acute rejection is defined by a combination of: (a) an acute rise in serum creatinine prompting a renal biopsy and (b) any pathology on the biopsy that meets the criteria for acute rejection, according to the latest BANFF criteria. The number of biopsies and the appropriate biopsy reports will be recorded on the CRF.

A full economic evaluation will adopt a National Health Service (NHS) perspective. The 3-year outcomes are the rates of: (1) graft failure, (2) patient survival, (3) graft dysfunction (see definition above), (4) acute rejection and (5) culture-positive infection, malignancy or diabetes, together with (6) EQ-5D, which is a patient-specific quality-adjusted life years (QALY) measurement. Cost-effectiveness will use (1) to (5) where (1) is the primary and the others are secondary outcomes. Cost-utility will be measured using (6) (the EQ-5D questionnaire). The net benefit per patient will be calculated by multiplying QALY by the assumed maximum willingness-to-pay for QALY (£20,000 per QALY) and subtracting the costs. The costs of all interventions will be obtained from Guy’s or estimated by identifying relevant categories of resource utilisation or measuring the volume of each category and multiplying by the average NHS resource costs [15] (and using British National Formulary and NHS reference lists). The costs of an intervention include the cost of screening beads and enhanced drug costs.

Participants’ reporting of adherence will be assessed using the Medicines Adherence Report Scale (MARS) [16,17], a valid and reliable scale that has been previously used to assess adherence in renal transplant recipients [18,19]. Self-report measures have the advantage of being inexpensive and non-intrusive. However, it is known that self-reporting underestimates the true extent of non-adherence because of inherent self-presentational and recall biases. Self-presentational bias occurs when respondents may be reluctant to admit to non-adherence because they perceive a social contract where the expectation is one of high adherence. MARS takes steps to diminish this bias by sanctioning and normalising reports of non-adherence. However, this does not totally remove the effect of self-presentational and recall biases that are inherent in all self-report measures. For this reason we will apply a combined approach to adherence assessment, where an initial categorisation of patients into high vs low on the basis of self-reporting is revised based on calcineurin inhibitor blood monitoring (carried out in routine management for patients prescribed tacrolimus or ciclosporin) and tablet counts (conducted for a sample of participants). In this approach, reports of low adherence are accepted as self-presentational biases that act in the opposite direction (reports of low adherence are more reliable than reports of high adherence [20,21]). Patients who report high adherence are reclassified to low adherence on the basis of calcineurin inhibitor trough results (for example, if levels are undetectable then the participant is assumed to be non-adherent) or tablet counts (for example, if there is a greater than 20% discrepancy between the actual and expected tablet count then the participant is reclassified as non-adherent).

To explore the potential antecedents to participants’ adherence behaviours, they will be also be asked to complete specially adapted versions of questionnaires relating to treatment intrusiveness, symptoms associated with immunosuppressants, beliefs about medicines [22], satisfaction with information about their medicines [23] and whether they are feeling anxious and/or depressed [24]. Questionnaires will be used to qualify perceptions of personal risk [25]. On the basis of survey responses, a small number of participants will be purposively selected (for example, those with positive and negative attitudes, and high and low adherers) for qualitative interview to explore their perception of risk and adherence behaviours in more depth.

All patients taking part in the trial will be asked to complete all or some of the questionnaires at specified times. Questionnaires will be administered electronically. Respondents will complete a survey online whilst in a clinic, using an iPad or equivalent tablet device designated solely for this trial. Completed survey responses are stored on Qualtrics secure servers and can only be accessed using a login and password. Nothing will be recorded on the main trial CRFs.

The questionnaires will be piloted with the first few participants recruited to the Guy’s site. These respondents will be asked to undergo cognitive interviewing whilst completing the survey, a technique used to ensure the validity of questionnaire items [26]. On the basis of this pilot, the questionnaire items may undergo minor modification. The ease of utility of the online survey and tablet device will also be evaluated during the pilot.

### Ethical approval

The OuTSMART trial has been approved by the National Research Ethics Service Committee London, Hampstead, (REC number 12/LO/1759 on 14 January 2013) and by the MHRA (EudraCT number 2012-004308-36 on 24 December 2012).

## Discussion

Research by others working in this area has mostly focussed on how to treat established CR. All reports consider small numbers of patients. Theruvath *et al*. reported 12-month stabilisation of kidney function in ¾ patients with kidney dysfunction due to CR associated with HLA Ab after transfer onto tac and MMF and a short course of prednisolone [27]. In addition, several studies have reported successful stabilisation of CR using B cell depletion therapy [28-30], further supporting the hypothesis that targeting underlying cellular responses, rather than HLA Ab, is a rational approach to treatment for these patients.

Several other strands of evidence support the use of an optimised tac and MMF regime to prevent progression of CR. First, both MMF [31] and tac [32] are better at suppressing acute rejection than alternative agents and the combination of the two agents achieves better outcomes at 1 and 2 years compared to alternative regimes [33,34]. Second, regimes containing MMF are associated with a lower prevalence of HLA Ab [35] and MMF specifically reduces the development of HLA Ab during episodes of acute rejection [36]. Third, although these benefits have not always fed through to improvements in graft survival rates, one recent landmark study did show improved graft survival for a combination of tac and MMF [37]. For tac, enhanced graft survival also emerged during a systematic Cochrane review comparing tac with ciclosporin [34]. For MMF, a retrospective analysis of United States registry data revealed an association with significantly lower rates of premature allograft failure [38]. Finally, some studies have shown that conversion from ciclosporin to tac is beneficial for patients with deteriorating graft function [39], and that introduction of MMF has a similar effect [40]. Although other studies have reported contradictory results [41], much of this literature is difficult to interpret because many studies do not distinguish between CR and other causes of chronic graft dysfunction [42].

In addition to tac and MMF, we propose to use a short course of moderate-dose prednisolone followed by low dose steroid maintenance in this trial. There is no direct evidence from the transplant literature to support this intervention, but a similar treatment course is standard therapy in many situations where quick and effective suppression of immune responses is required, for example in acute asthma and in many types of autoimmune diseases.

In summary, this trial will identify a group of patients at high risk of premature transplant failure. Therapy will be optimised for these at-risk patients, many of whom will be identified before they have started to deteriorate clinically. As described above, the natural history of untreated CR is of progressive loss of function, usually at a predictable rate, leading eventually to complete loss of graft function. Depending on the initial creatinine level, the time to graft loss will be variable, but graft loss rarely occurs without this period of progressive loss of function. Lachmann *et al*. [8] reported a constant rate of graft loss from the start of their study, so we expect that the rate of graft loss in our study will be constant in the standard care group. We predict that optimised immunotherapy will change the natural history of the condition, and lead to stabilisation of function in a significant proportion (50%) of those with HLA Ab. This will be visible in our analyses of the secondary end points. Hopefully the treatment will prevent the predicted graft loss in the first 3 years for this group and thus impact on the primary end point of the study.

## Trial status

At the time of submission, the study has a planned recruitment start date of 27 August 2013.

## Endnotes

^a^In the transplantation literature, this problem is called ‘late’ allograft failure, in which ‘late’ refers to the lifespan of the transplanted organ. We have changed the term to shift the emphasis to the recipient.

## Abbreviations

Ab: antibodies; BLC: biomarker-led care; CR: chronic rejection; DM: diabetes mellitus; DSA: donor-specific antibodies; eGFR: estimated glomerular filtration rate; EME: efficacy and mechanism evaluation; GFR: glomerular filtration rate; HIV: human immunodeficiency virus; HLA: human leukocyte antigen; IgG: immunoglobulin G; IMP: investigational medical product; KCTU: King’s Clinical Trials Unit; MARS: Medicines Adherence Report Scale; MDRD: modification of diet in renal disease; MFI: mean fluorescence intensity; MHRA: Medicines and Healthcare Products Regulatory Agency; MMF: mycophenolate mofetil; NIHR: National Institute for Health Research; NHS: National Health Service; PBMC: peripheral blood mononuclear cells; PCR: protein/creatinine ratio; PI: principal investigator; PIN: personal identification number; QALY: quality-adjusted life years; SAB: single-antigen beads; SC: standard care; tac: tacrolimus.

## Competing interests

The authors declare no competing interests.

## Authors’ contributions

AD is the chief investigator of the study and wrote the paper. Rac H, RB, RJ, MP and RB are co-PIs and will be recruiting patients. RV, BC, MB, SM and DB are co-PIs and will be responsible for tissue typing and HLA testing. IRM and JLP are the trial statisticians. LG is the trial manager. GD helped with writing and will be recruiting patients. Rob H will perform the assessment of adherence and risk analysis. PMcC will perform the health economic analysis. JK is the database manager. CM is the Operational Director of KCTU at King’s Health Partners. All contributed to the trial design and reviewed the paper. All authors read and approved the final manuscript.
